# Identifying key priorities for research to protect the consumer with food hypersensitivity: A UK Food Standards Agency Priority Setting Exercise

**DOI:** 10.1111/cea.13983

**Published:** 2021-07-23

**Authors:** Paul J. Turner, Elizabeth Andoh‐Kesson, Sarah Baker, Alexa Baracaia, Alisha Barfield, Julie Barnett, Karen Brunas, Chun‐Han Chan, Stella Cochrane, Katherine Cowan, Mary Feeney, Simon Flanagan, Adam T. Fox, Leigh George, M. Hazel Gowland, Christina Heeley, Ian Kimber, Rebecca Knibb, Kirsty Langford, Alan Mackie, Tim McLachlan, Lynne Regent, Matthew Ridd, Graham Roberts, Adrian Rogers, Guy Scadding, Sarah Stoneham, Darryl Thomson, Heidi Urwin, Carina Venter, Michael Walker, Rachel Ward, Ross A. R. Yarham, Maggie Young, John O’Brien

**Affiliations:** ^1^ National Heart & Lung Institute Imperial College London London UK; ^2^ British Retail Consortium London UK; ^3^ Anaphylaxis Campaign Farnborough UK; ^4^ Imperial College London London UK; ^5^ Food Standards Agency London UK; ^6^ Department of Psychology University of Bath Bath UK; ^7^ Unilever Safety and Environmental Assurance Centre (SEAC Sharnbrook UK; ^8^ Katherine Cowan Consulting East Sussex UK; ^9^ King's College London Guy's and St Thomas’ NHS Foundation Trust London UK; ^10^ Reading Science Centre Mondelēz International UK; ^11^ Allergy UK Sidcup UK; ^12^ Allergy Action St Albans UK; ^13^ Barnsley Metropolitan Borough Council Barnsley UK; ^14^ Aston University Birmingham UK; ^15^ Bath and North East Somerset Council Bath UK; ^16^ University of Leeds Leeds UK; ^17^ Natasha Allergy Research Foundation Isleworth UK; ^18^ Population Health Sciences University of Bristol Bristol UK; ^19^ Clinical and Experimental Sciences Academic Unit Faculty of Medicine University of Southampton Southampton UK; ^20^ Romer Labs UK, Ltd Runcorn UK; ^21^ Royal Brompton & Harefield Hospital NHS Trust London UK; ^22^ Food Experts Group UK Hospitality London UK; ^23^ Coeliac UK High Wycombe UK; ^24^ Section of Allergy/Immunology Children's Hospital Colorado and University of Colorado Denver CO USA; ^25^ Office the Government Chemist Teddington UK; ^26^ Exponent International Ltd Harrogate UK; ^27^ Nutrition Innovation Centre for Food & Health School of Biomedical Sciences Ulster University Coleraine UK

**Keywords:** allergen labelling, coeliac disease, food allergy, James Lind Alliance, research prioritization

## Abstract

**Introduction:**

Food hypersensitivity (FHS), including food allergy, coeliac disease and food intolerance, is a major public health issue. The Food Standards Agency (FSA), an independent UK Government department working to protect public health and consumers’ wider interests in food, sought to identify research priorities in the area of FHS.

**Methods:**

A priority setting exercise was undertaken, using a methodology adapted from the James Lind Alliance—the first such exercise with respect to food hypersensitivity. A UK‐wide public consultation was held to identify unanswered research questions. After excluding diagnostics, desensitization treatment and other questions which were out of scope for FSA or where FSA was already commissioning research, 15 indicative questions were identified and prioritized by a range of stakeholders, representing food businesses, patient groups, health care and academia, local authorities and the FSA.

**Results:**

295 responses were received during the public consultation, which were categorized into 70 sub‐questions and used to define 15 key evidence uncertainties (‘indicative questions’) for prioritization. Using the JLA prioritization framework, this resulted in 10 priority uncertainties in evidence, from which 16 research questions were developed. These could be summarized under the following 5 themes: communication of allergens both within the food supply chain and then to the end consumer (ensuring trust in allergen communication); the impact of socio‐economic factors on consumers with FHS; drivers of severe reactions; mechanism(s) underlying loss of tolerance in FHS; and the risks posed by novel allergens/processing.

**Discussion:**

In this first research prioritization exercise for food allergy and FHS, key priorities identified to protect the food‐allergic public were strategies to help allergic consumers to make confident food choices, prevention of FHS and increasing understanding of socio‐economic impacts. Diagnosis and treatment of FHS was not considered in this prioritization.

**Key messages**

- We undertook a food hypersensitivity research priority setting exercise for the UK Food Standards Agency.
- This was informed by a public consultation and multiple stakeholder input, but excluded diagnostics and treatment
- People with food hypersensitivity identified issues around making confident food choices as key research priorities.

## 1 INTRODUCTION

Food hypersensitivity (FHS)—a term which encompasses food allergy (both IgE‐mediated and non–IgE‐mediated mechanisms), coeliac disease and food intolerances—continues to be a major issue in terms of the supply of safe food for consumers. According to the UK’s Food Standards Agency (FSA), around 1 in 20 of the UK population report a FHS. Food allergy is the commonest cause of potentially life‐threatening allergic reactions (anaphylaxis), a serious systemic hypersensitivity reaction that is usually rapid in onset and may cause death.^1^ Hospital admissions due to food‐anaphylaxis continue to increase in the UK and elsewhere, although reassuringly, the case fatality has not increased.^2^, ^3^

The provision of safe food to consumers with FHS is a major priority for the FSA, an independent Government department working to protect public health and consumers’ wider interests in food. The FSA has been a significant funder of FHS‐related research in the UK.^4^ The FSA's Board requested its Science Council, a group of independent scientific advisers, to undertake a review of its research programme on FHS in 2019 and help inform its future direction in terms of commissioning research.

Research priorities are often developed without wide and coordinated stakeholder contributions.^5^ To address this concern, the James Lind Alliance (JLA) has developed a methodology for Priority Setting Partnerships (PSPs), which brings together patients, carers and clinicians to identify and prioritize the evidence uncertainties in any given topic area.^6^ The aim of a PSP is to help ensure that those who fund health research are aware of what really matters to patients, carers and clinicians, the end users of research. The JLA uses an adapted nominal group technique; this prevents the domination of discussion by a single person and encourages the participation of less assertive members, including non‐professionals. PSPs have been undertaken for numerous chronic diseases including asthma^7^ and eczema^8^ but not for food allergy or food hypersensitivity. Industry stakeholders and those with commercial interests are usually excluded from PSPs. However, in context of the FSA’s remit to protect consumers with respect to risks posed by FHS, it was essential to include Food Business Operators and other stakeholders involved in the food supply chain. Therefore, in order to meet the requirements of the FSA, a research Priority Setting Exercise (PSE) was undertaken, adapting PSP principles with the inclusion of industry stakeholders, to identify research priorities for the FSA in the area of FHS.

The aim of the PSE was to identify and prioritize the current knowledge gaps in providing safe food to individuals with FHS in the UK from key stakeholder perspectives, including (but not limited to) consumers (both allergic and non‐allergic), healthcare professionals, regulators, industry and wider stakeholders. The scope of the PSE included the following: enabling safe food choices for consumers with FHS; practises to handle and produce food safely for those with FHS; and behaviours surrounding food safety with specific reference to FHS. Although the scope of the PSE did not include underlying health delivery (including access to diagnosis and treatment of FHS) as these are not within the FSA’s remit, information regarding these issues were collected and reported here, although not included in the prioritization exercise. In reporting this exercise, we have referred to the REPRISE Reporting guideline for priority setting of health research.^9^

## 2 METHODS

**FIGURE 1:**
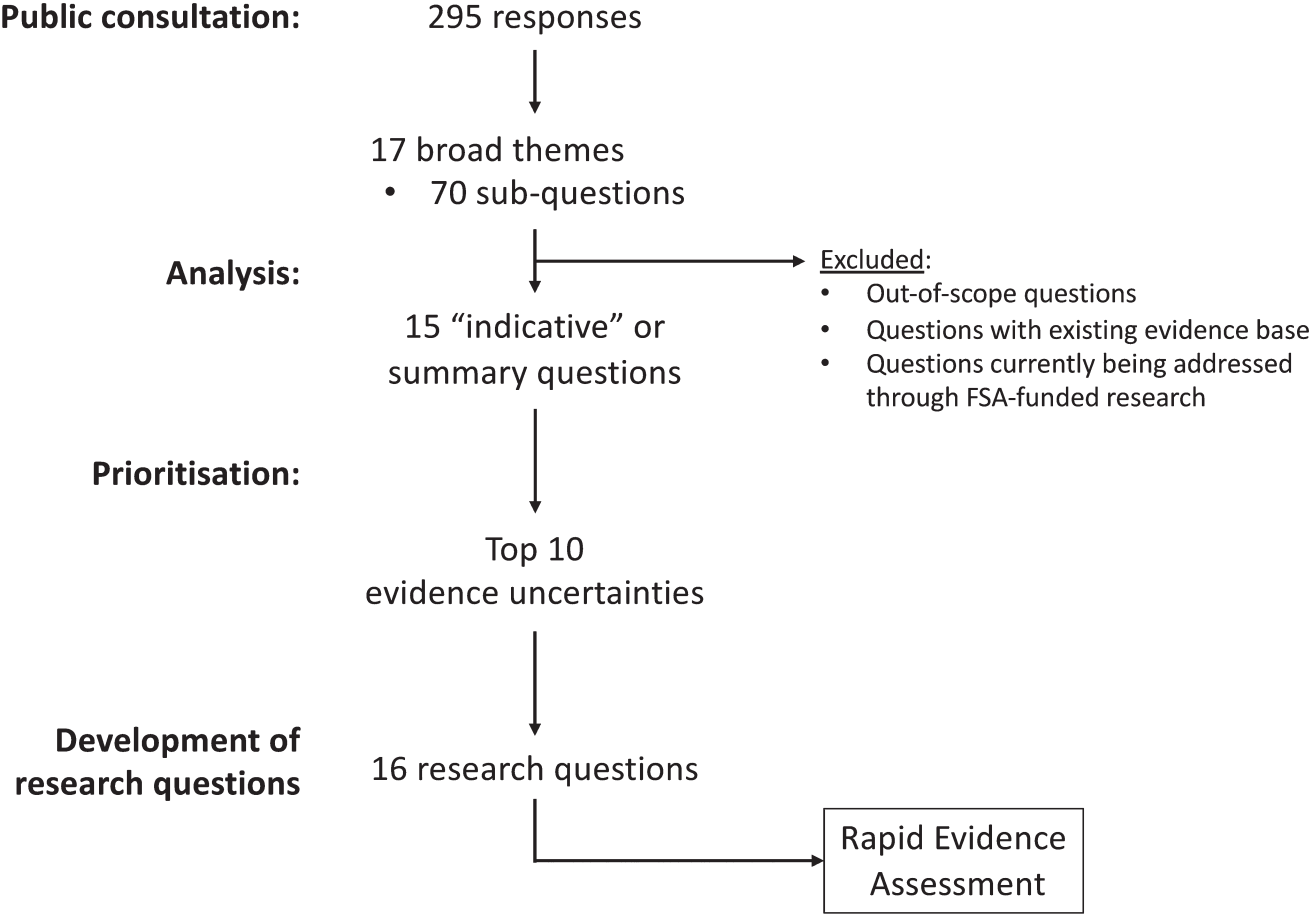
Outline of methodology

There were 5 stages to the PSE, outlined as follows and in Figure 1:

- *Initiation and identification of potential stakeholders*: a Steering Group made up of members of the FSA Science Council and Secretariat was established to write a protocol,^10^ oversee the PSE activity and identify potential stakeholders. The protocol was aligned with JLA methodology as per the JLA Guidebook^6^ and finalized in December 2019.
- *Identifying knowledge gaps*: A UK‐wide, online consultation of public stakeholders was undertaken to identify ‘unanswered questions’ and knowledge gaps (referred to as ‘evidence uncertainties’) relating to the provision of safe food to consumers with FHS.
- *Analysis and formulation of research questions*: refinement of responses generated in (ii) to formulate summary questions. This work was contracted to Ipsos MORI and overseen by the PSE Steering Group.
- *Prioritization workshop*, where the summary questions identified in (iii) were prioritized through consensus, with the input from representatives of the various stakeholder groups, held in September 2020.
- *Development of research questions* based on the identified priorities, to help the FSA understand the existing evidence base and thus the need for future research.

### 2.1 Identification of knowledge gaps and public consultation

Five themes were identified by the Steering Group to provide structure to the PSE:

- *Eating out*: the consumption of food prepared and served away from home, especially at a restaurant, café or take away establishment.
- *Buying prepacked food* that is food that has been prepared in advance of sale, for example ready meals and packaged sandwiches.
- *Handling and Understanding Food*—helping consumers to make informed choices about buying safe food, which involves the following: food preparation, labelling, food/ingredient supply, preventing cross‐contamination, effective cleaning, testing and monitoring to ensure food safety.
- *How we interact with food*, including changes in how and where consumers obtain food today, for example new foods and novel allergens, food banks, food business practices, new, reusable and biodegradable packaging and online purchasing through the Internet.
- *Improving knowledge* including, questions about the numbers of people in the UK affected by food hypersensitivity; or why some people develop food hypersensitivity but then outgrow their allergy or sensitivity.

**FIGURE 2:**
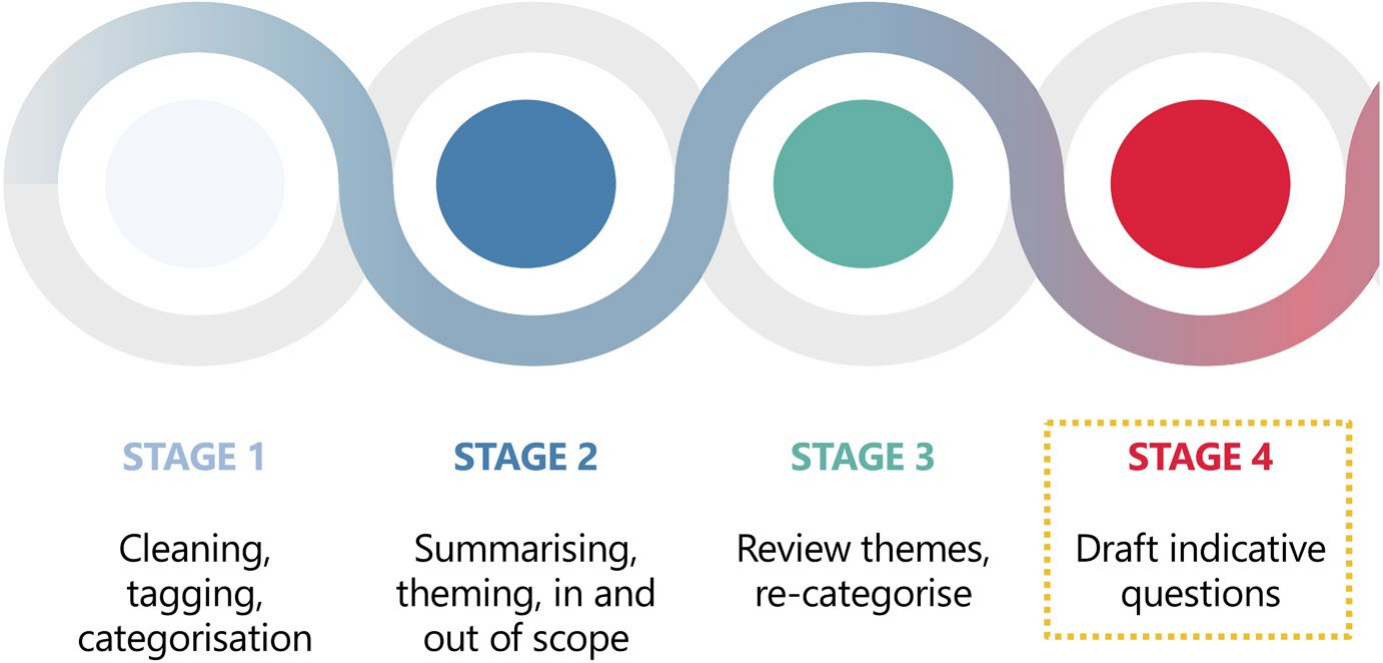
Analysis of responses to the public consultation into indicative questions for the PSE. Further details available in the Ipsos MORI report^10^

An online public consultation ‘Improving life for people with Food Hypersensitivity’ was created using Microsoft Teams and launched on 20 February 2020 (see supporting information). The public consultation was communicated to over 250 organizations *via* social media channels, targeting the general public, food businesses, patient groups/charities, religious and cultural organizations, the healthcare sector, academia, local authorities and professional bodies. Respondents were asked to help identify knowledge gaps relating to FHS in each of the above 5 themes. Responses from the UK were automatically collected in Microsoft Excel, cleaned and then transferred to Ipsos MORI who were commissioned to analyse the responses received, using a 4‐stage process as shown in Figure 2.

After excluding out‐of‐scope questions or those areas in which FSA is currently commissioning research, the Steering Group developed 15 indicative questions to capture the key themes and concerns raised in the public consultation. These were then taken to a two‐day prioritization workshop in September 2020 (virtual meeting, postponed from early 2020 due to COVID) conducted according to the JLA Guidebook.^6^ Thirty‐two stakeholders, representing food businesses, patient groups, healthcare professionals and academia, local authorities and the FSA, were present. The workshop followed the standard JLA workshop method, drawing on an adapted version of Nominal Group Technique and consisting of a rounds of mixed‐stakeholder group discussions and ranking exercises. The workshop was facilitated by independent facilitators with experience in the JLA priority setting method. Relevant research questions relating to the identified priority indicative uncertainties were then developed by the same attendees at a subsequent workshop. A standard pro forma, based on a PICO format (Participants, Intervention, Comparator, Outcome), was used to guide discussions. No reimbursement was provided for participation.

## 3 RESULTS

**FIGURE 3:**
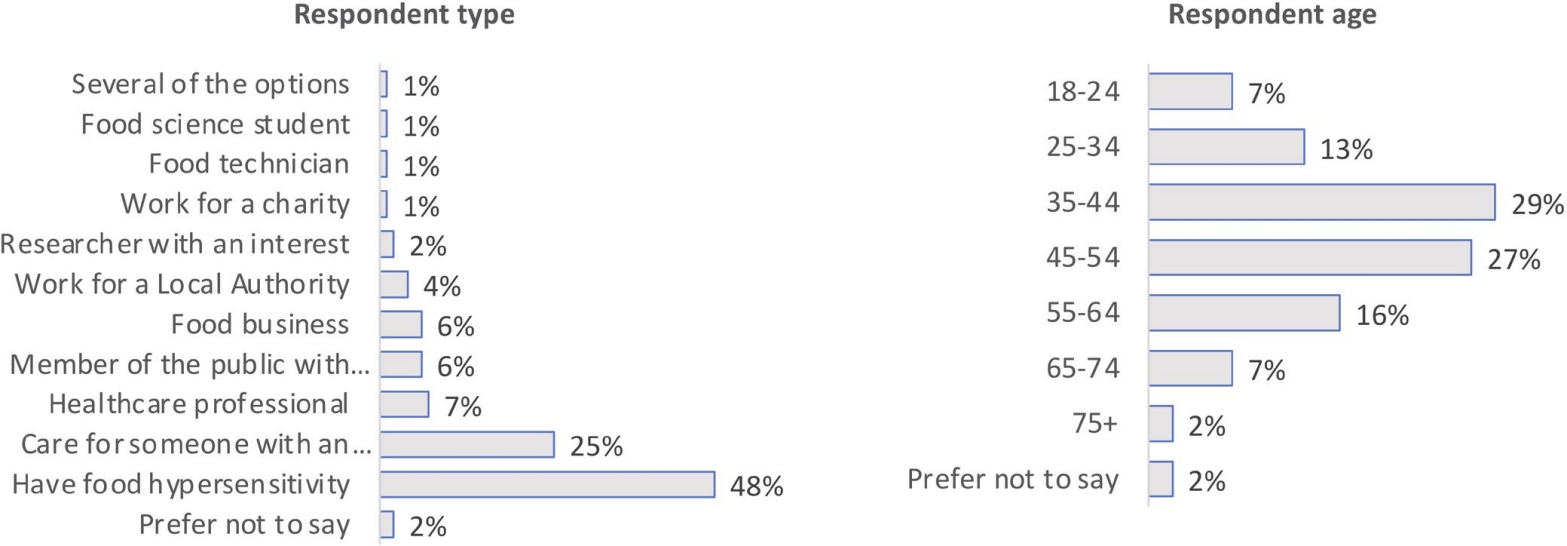
Respondents to the public consultation

The public consultation received 295 responses by the time the survey closed (a month earlier than anticipated, due to COVID‐19) on 26 March 2020, representing a broad group of stakeholders (Figure 3), 96% of whom were based in the UK. Responses were categorized according to ‘tags’ (Table 1) which were then used to identify more specific sub‐questions. In total, 17 broad themes were generated, with 70 sub‐questions within these themes (see Supporting information: Table S1; further details appear in the Ipsos MORI report available online^11^). Very broadly the questions and issues identified by different stakeholders were similar, although specific types of respondents had slightly greater interest in certain issues.

- Healthcare professionals—allergen contamination (eg due to the use of shared production lines); communication of (potential) allergen presence to consumers, including through labelling and precautionary allergen (‘may contain’) labels; knowledge and understanding of FHS; and science underlying the development or loss of tolerance resulting in FHS.
- Food business operators (FBOs)—knowledge and understanding of FHS; provision of allergen information between FBOs within the supply chain and to the end consumer.
- Patients with FHS and their carers—diagnosis and treatment of FHS; making safe food choices, including when eating out; measures to minimize contamination during production; and communication of allergen information including precautionary allergen labels.

**TABLE 1:** Main themes identified during the public consultation

Themes	Mentions (n)	Examples of tags in each theme
Packaging and labelling	658	Comprehensive, clear, disclaimers, packaging, allergens, gluten
Allergic consumers	631	Reactions, hypersensitivity, number, frustrations, diagnosis, treatment, Irritable Bowel syndrome, coeliac disease
Cross‐contamination	397	Manufacturing, processing, products on display, staff behaviour
Information	364	Ingredient lists, digital info, clarity, allergen list
Knowledge / education	306	Better training, guidance, understanding, take it serious, allergy vs. intolerance
Safety	325	Tolerance levels, levels of risk, trust
Allergens	311	14 EU Priority allergens, rapeseed, additives, egg, cow's milk (dairy), nuts
Food	258	Prepacked, more choice, novel food, vegan, vegetarian
Legislation / standardization	239	Ratings, testing, reporting, FSA
Eating out	202	Food preparation, menus, staff knowledge

The Steering Group then reviewed these sub‐questions, and after excluding out‐of‐scope questions or those areas in which FSA is currently commissioning research (Table 2), 15 indicative questions were developed (see supporting information, Table S2) and taken forwards for prioritization at the PSE workshop in September 2020 (virtual meeting, postponed from early 2020 due to COVID). Using the JLA prioritization framework, this resulted in 10 priority indicative uncertainties, from which 16 research questions were developed (Table 3).

**TABLE 2:** Excluded evidence uncertainties due to out of scope or through being addressed by ongoing FSA‐contracted research

Out of scope	Addressed by current FSA research
What is the difference between an allergy and an intolerance?	How many people are affected by FHS?
Why do healthy eating options include so many allergens?	How many hospital/GP visits are due to FHS?
Gluten‐free foods: Why do they cost more? Are they ‘better’ for you?	What are the most common food allergies/intolerances? Is this changing?
Diagnostics: Waiting times, accuracy, access, novel diagnostics and genetics	National register or database for allergy incidents/people with FHS
Desensitization treatment for food allergy, including interventions targeting the microbiome	Are food allergies/intolerances increasing?
Are staff in food establishments trained in how to use adrenaline autoinjector devices?	Is there a link between childhood eczema and food allergy?
Are food allergies in adults treated with the same seriousness as those in children?	Thresholds for clinical reactivity, that is how much allergen is needed to trigger reactions
Is there a link between food poisoning and the development of FHS?	
What is the defined safe level of lead in game birds?	

**TABLE 3:** ‘Top 10’ Research questions identified by the PSE

Indicative uncertainty	Suggested research question(s)	Notes
Risks posed to people with FHS by new/novel foods and/or processes	In consumers with FHS, what measures are needed to monitor for reactions due to: new uses of known allergens? novel proteins which might induce sensitization and thus clinical reactivity? What protocols should the FSA use when assessing the risk to consumers with FHS posed by novel foods/processes/packaging? What data exist as to the likelihood of allergenic proteins in biobased food contact materials migrating into foods?	For example, the use of pea protein in protein concentrates, which is often declared only as ‘vegetable protein’ in ingredients listing. For example, wheat‐based starch in packaging, latex‐based binders in packaging and sustainable cutlery.
Improving traceability of allergens in the food supply chain	How should information be communicated (through the food supply chain) to consumers with FHS, to: improve consumer confidence in terms of possible allergen content? reduce the incidence of unintended allergen exposure?	The sensitivity and reliability of analytical tests was also discussed, but development of these and the responsibility to ensure such tools are used appropriately was felt to be outside the FSA’s research remit.
Risks posed due to shared production of foods, and how can these be mitigated	What are the health risks to consumers with FHS due to allergen cross‐contamination during food production? How effective are different control options (such as cleaning protocols) in reducing these health risks?	For example, the use of shared ovens (eg gluten‐free foods cooked in the same oven as gluten‐containing foods)
Communicating risk, so that consumers with FHS can be confident that the food they are provided is safe	What are the most effective ways for FBOs to communicate a level of competence (with respect to allergen risk management) to consumers?	
Allergen labelling, including Precautionary Allergen (‘may contain’) labels.	What forms of allergen labelling are effective in order for consumers to make informed decisions as to whether a food is ‘safe’ for purchase/consumption?	Labelling to inform both what is present, what might be present (through cross‐contamination), and what is not present (whether or not a ‘free‐from’ claim is made).
Informing the FSA as to incidents involving food hypersensitivity.	What evidence is there for different reporting systems to deliver useful data to regulators that can impact on reducing the risk of unintended allergen consumption? What are the barriers that prevent reporting of near misses and other incidents to official bodies?	Reporting systems might include the following: Mandatory/voluntary reporting by healthcare professionals. Direct reporting by FBOs and the public Surveillance of serious incidents e.g. coronial system
Impact of co‐factors on reaction severity	In consumers with FHS, what are the factors which can increase the risk of a severe reaction? How should risk posed by co‐factors be communicated to those affected by FHS?	Incorporates both general advice to all consumers with FHS, and individualised advice provided by healthcare professionals
Impact of socio‐economic factors (including race/ethnicity) on FHS	What are the socio‐economic factors which impact on risk in consumers with FHS? How do cultural attitudes impact on the management of FHS?	Includes the following: understanding the impact of ethnicity/race as confounders language impacting on access to effective advice and communication of consumer needs impact on affordability/ accessibility/availability to safe foods for those with FHS
Impact of environmental exposures on risk of developing FHS	What are the factors that drive a loss of immune‐tolerance to food allergens? (Discussion was also held with respect to the impact of disturbances in the microbiome affecting risk of FHS, but it was considered that funding research in this area would be beyond the scope of the FSA)	Applies to both childhood‐ and adult‐onset allergy, for example how common is loss of prior tolerance?
Current knowledge of FHS amongst the general public	What are the current gaps/inaccuracies in knowledge with respect to FHS amongst the general public?	Focus on general public, but also applies to specific stakeholders, for example FBOs, health care

## 4 DISCUSSION

The JLA methodology was originally developed to bring patients and clinicians together in a non‐hierarchical manner to identify and address the most important uncertainties with respect to the effects of care and treatments.^12^ It has since been used to prioritize questions for research in over 100 clinical areas internationally. We report the outputs from a prioritization exercise using this methodology to highlight priority unanswered questions in terms of providing safe food to consumers with FHS. This is the first research prioritization exercise ever conducted for food allergy and FHS. A major strength is the very significant and comprehensive input from patients/consumers achieved as a result of the initial public consultation. Although the focus was on UK consumers, it is likely that many (if not all) of the identified indicative uncertainties would apply elsewhere. The research questions proposed are not intended to be exhaustive, but, rather, reflect the views of the workshop participants in terms of areas where addressing the underlying evidence uncertainties could help better provide for consumers with FHS. The traditional JLA approach ends with the identification of priorities. These tend to be broad topic areas which require interpretation and development to become tractable propositions for research. We have built on this process by incorporating that additional step into the project, to identify specific research questions from the priorities, involving multiple stakeholders to ensure those questions genuinely reflect the concerns of the evidence users.

Healthcare delivery, including therapeutics, are outside the FSA’s remit and were therefore considered out of scope. However, the public consultation demonstrated that these remain priorities for consumers with FHS, and we therefore list these unanswered questions in Table 2. A need to improve diagnostics (accuracy, accessibility) and develop new therapeutics for FHS (including, but not limited to allergen desensitization and interventions targeting the microbiome) are highlighted. Given that respondents were encouraged not to consider issues relating to healthcare provision when completing the public consultation, the list of out‐of‐scope priorities should not be considered exhaustive, and a future PSP focussed on food allergy management and treatment is justified. We attempted as broad a public consultation as possible, using specific targeting of community charities and religious organizations to capture the inclusion of consumers who might otherwise be excluded, for example children/young people and minority groups. Despite this, it is possible that some groups may have been under‐represented. In this respect, it will be interesting to compare the outputs of the ongoing COMFA (Core Outcome Measures for Food Allergy) project which seeks to develop a set of Core Outcomes for evaluating new treatments for food allergy.^13^

The priorities identified fit into 5 themes. First, communication of allergens both within the food supply chain (between different FBOs) and then to the end consumer (ensuring trust in the allergen information available); (ii) the impact of socio‐economic factors on both the development of FHS and then the risk of more severe reactions; (iii) other drivers of severity; (iv) the mechanism(s) by which individuals can develop FHS to a previously tolerated food (something which is increasingly reported with respect to adult‐onset FHS); and (v) the risks posed by novel allergens/processing, something which is becoming more important given the increasing global population and the desire towards sustainability. There are many similarities between these themes and those identified by PSPs for other related conditions, including asthma, eczema and coeliac disease.^7^, ^8^, ^14^ For coeliac disease, risk communication by FBOs (including allergen labelling), diagnostic accuracy, increased awareness amongst the general public have also been highlighted as priority areas.^14^

Some of these unanswered questions can be addressed through improving the surveillance of FHS reactions occurring in the community, to inform both current policy and allow the detection of new allergen risks (either arising from novel allergens/processes, or changes in the consumption patterns of existing allergens) which pose a hazard to consumers with FHS. In this respect, the FSA is funding the establishment of a UK‐wide Anaphylaxis Registry to report allergic reactions and developing a Food Allergic Reaction Reporting Mechanism to better capture food incidents from FBOs and the general public.^15^

The communication of allergen risk through the food supply chain and to the end consumer is partly informed by legislation. The public consultation was undertaken prior to the COVID‐19 epidemic, which impacted upon the food supply chain; changes in the availability of ingredients and the increased use of online ordering and dark kitchens have demonstrated potential vulnerabilities which could impact on the efficacy of allergen communication. The interpretation of precautionary allergen (‘may contain’) labels (PAL) remains a concern to consumers, FBOs and regulators alike.^16^ PAL are not specifically informed by legislation at the current time (although the use of PAL should not be misleading under the General Food Law). Work is currently ongoing at a global level to reassess the evidence base for use of PAL.^17^ This should improve scientific rigour and transparency to the communication of risk posed by unintended allergen presence (eg due to cross‐contamination during food production), allowing consumers with FHS to make more informed and safer food choices.

There is a clear impact of socio‐economic factors on food allergy in the USA^18^ but the extent to which this may be true in the UK is unclear. A London‐based study reported a relatively greater increase in children diagnosed with peanut allergy over a 14‐year period (1990–2004) from non‐white backgrounds, compared to egg allergy, which could not be explained by improved awareness.^19^ Another study has reported a significantly higher incidence of anaphylaxis amongst British South Asians compared with those from a White Caucasian background.^20^ In the EAT study, authors noted a much higher rate of food allergy in non‐white participants, from 5.3% in those from a white background to 19.3% in Asian/black/Chinese participants.^21^ The EAT study also indicated that cultural norms and communication may be a factor, with adherence to advice lower in those from a non‐white background.^22^ The relationship between ethnicity and socio‐economic class can be difficult to unpick and can also impact on access to health care and communication (which is important in terms of dietary advice and risk avoidance). The PSE has identified this as a major knowledge gap which should be addressed to help resolve the disparities that appear to exist with respect to ethnicity and FHS.

Creating a research agenda involves many different drivers: for many chronic diseases, research direction is affected by commercial interests and the ‘patient voice’ is something which may not figure prominently; it is this inequality that the JLA methodology for PSPs was designed to address.^5^ At the same time, the absence of ‘treatments’ for FHS (with management focussed more on avoidance of the trigger and rescue medication in the event of accidental reactions) creates different drivers compared with other chronic conditions. Legislation and regulation set a minimum standard for FBOs to adhere to, but consumers may not have confidence and trust that their needs are being provided for, for instance with respect to the purchase of foods from a catering outlet or the use PAL. An FBO can be fully compliant with legislation and guidance, yet consumers may not feel confident that the FBO can provide them with ‘safe’ food, for example, due to poor communication. Improving this requires a combination of ‘science push’ and ‘policy pull’, with clear and transparent communication to consumers so that they can develop trust in FBOs providing food for them. In undertaking this exercise, we hope to have identified key areas where further research and funding should be targeted, from the perspectives of the various stakeholders who will be the beneficiaries and end users of FHS research. The hope is that this will better provide for the significant proportion of the population with FHS and reduce the day‐to‐day impact of a diagnosis of FHS on patients.

## CONFLICT OF INTEREST

All authors have completed the ICMJE uniform disclosure form at www.icmje.org/coi_disclosure.pdf and declare competing interests as follows: PJT reports grants from the Food Standards Agency, JM Charitable Foundation, Medical Research Council, NIHR/Imperial Biomedical Research Centre and End Allergies Together, outside the submitted work; personal fees from UK Food Standards Agency, Aimmune Therapeutics, AllerGenis and ILSI Europe outside the submitted work. JB reports personal fees and grants from Food Standards Agency, outside the submitted work. SC reports financial activities with Unilever, outside the submitted work. KC is an independent facilitator and senior adviser to the James Lind Alliance. She is co‐author/editor of the JLA Guidebook and supports the JLA Secretariat and team of JLA advisers. She was an independently contracted adviser for this work. MF reports personal fees from Aimmune Therapeutics and Danone outside the submitted work. ATF is current President of the British Society for Allergy & Clinical Immunology (BSACI) and Chair of the Health Advisory Board of Allergy UK, both of which receive corporate sponsorship from companies involved in food allergy. He also reports consultancy work with Aimmune, without receiving a personal fee. MHG reports personal fees from Food Standards Agency (as a subcontractor), outside the submitted work. IK reports personal fees from Food Standards Agency, outside the submitted work. J’OB John O'Brien is a trustee of the Institute of Food Science & Technology, a member of the non‐executive board of Campden BRI, and Director of the Food Observatory; personal fees from Food Standards Agency, outside the submitted work. GR reports grants from European Union and Food Standards Agency outside the submitted work; he is also President Elect of the BSACI. CV reports grants from National Peanut Board; grants and personal fees from Reckitt Benckiser; personal fees from Nestle Nutrition Institute, Danone, Abbott Nutrition, INTENT Study, and Else Nutrition, outside the submitted work. The other authors do not report any conflicts of interest.

## AUTHOR CONTRIBUTION

PJT developed the concept and initiated the prioritization exercise (PSE) reported in this manuscript, on behalf of the UK Food Standards Agency. The PSE and related activities were coordinated by PJT, AB, CC, KC and JO'B. All authors participated in the PSE and subsequent workshops. PJT wrote the initial draft, which was subsequently reviewed, revised and the final version approved by all authors.

## Supporting information

Supplementary Material

## Data Availability

The data that support the findings of this study are available from the corresponding author upon reasonable request. The data are not publicly available due to privacy restrictions.
