# Supplementary material for: Identifying key priorities for research to protect the consumer with food hypersensitivity: A UK Food Standards Agency Priority Setting Exercise
Source: Clin Exp Allergy. 2021 Jul 23;51(10):1322–30. doi: 10.1111/cea.13983 (PMC9291826; doi:10.1111/cea.13983)
Supplement: Supplementary file 1 — Supplementary Material [file CEA-51-1322-s001.pdf]

Identifying key priorities for research to protect the consumer with food hypersensitivity: a UK Food Standards Agency Priority Setting Exercise

Paul J. Turner,[1] Elizabeth Andoh-Kesson,[2] Sarah Baker,[3] Alexa Baracaia,[4] Alisha Barfield,[5] Julie Barnett,[6] Karen Brunas,[4] Chun-Han Chan,[5] Stella Cochrane,[7] Katherine Cowan,[8] Mary Feeney,[9] Simon Flanagan,[10] Adam Fox,[9] Leigh George,[11] M. Hazel Gowland,[12] Christina Heeley,[13] Ian Kimber,[5] Rebecca Knibb,[14] Kirsty Langford,[15] Alan Mackie,[16] Tim McLachlan,[17] Lynne Regent,[3] Matthew Ridd,[18] Graham Roberts,[19] Adrian Rogers,[20] Guy Scadding,[21] Sarah Stoneham,[4] Darryl Thomson,[22] Heidi Urwin,[23] Carina Venter,[24] Michael Walker,[25] Rachel Ward,[26] Ross Yarham,[5] Maggie Young,[11] John O'Brien[27]

SUPPORTING INFORMATION

[1]National Heart & Lung Institute, Imperial College London, London, UK;
[2]British Retail Consortium, London, UK;
[3]Anaphylaxis Campaign, Farnborough, UK;
[4]Patient advocate, c/o Imperial College London, London, UK;
[5]Food Standards Agency, London, UK;
[6]Department of Psychology, University of Bath, Bath, UK;
[7]Unilever Safety and Environmental Assurance Centre (SEAC), Colworth Science Park, MK44 1LQ, Sharnbrook, UK;
[8]Katherine Cowan Consulting, East Sussex, UK;
[9]King's College London, & Guy's and St Thomas' NHS Foundation Trust, London, UK;
[10]Reading Science Centre, Mondelēz International, UK;
[11]Allergy UK, Sidcup, UK;
[12]Allergy Action, St Albans, UK;
[13]Barnsley Metropolitan Borough Council, Barnsley, UK;
[14]Aston University, Birmingham, UK;
[15]Bath and North East Somerset Council, Bath, UK;
[16]University of Leeds, Leeds, UK;
[17]Natasha Allergy Research Foundation, Isleworth, UK;
[18]Population Health Sciences, University of Bristol, Bristol, UK;
[19]Clinical and Experimental Sciences Academic Unit, University of Southampton Faculty of Medicine, Southampton, UK;
[20]Romer Labs UK, Ltd, Runcorn, UK;
[21]Royal Brompton & Harefield Hospital NHS Trust, London, UK;
[22]Food Experts Group, UK Hospitality, London, UK;
[23]Coeliac UK, High Wycombe, UK;
[24]Section of Allergy/Immunology, Children's Hospital Colorado and University of Colorado, Denver, USA;
[25]Office the Government Chemist, Teddington, UK;
[26]Exponent International Ltd., Harrogate, UK;
[27]Nutrition Innovation Centre for Food & Health, School of Biomedical Sciences, Ulster University, N. Ireland, UK.

Public stakeholder survey for Research Prioritisation Exercise

FSA Survey: Improving life for people with Food Hypersensitivity

The FSA is an independent Government department working to protect public health and consumers' wider interests in food. We make sure that food is safe and what it says it is.

We want the UK to become the best place in the world for people living with food hypersensitivities.

Do you have big questions that we could answer through research, to make things better for people with food hypersensitivity? Tell us your thoughts and help us to make a difference.

Why is the Food Standards Agency (FSA) carrying out this survey?

Around 2-3% of adults and 8% of children in the UK have a food hypersensitivity. This includes:

- food allergies (which involve the immune system, and can cause severe allergic reactions (anaphylaxis)
- coeliac disease, which affects about 1% of the UK population
- food intolerances (e.g. lactose intolerance) which do not involve the immune system).

We are carrying out this survey to get a better understanding about the key questions and issues the FSA needs to address through research, in order to better provide safe food for people with food hypersensitivities.

Who is the survey for?

- You can complete the survey if you are aged 18 years or over and you are:
- a member of the public with an interest in food hypersensitivity
- affected by food hypersensitivity yourself, or care for someone else with a food hypersensitivity
- a food business operator, representative or member of staff who has an interest in food hypersensitivity
- a charity representative or worker with an interest in food hypersensitivity
- a healthcare worker or researcher with an interest in food hypersensitivity
- a local authority or professional body with an interest in food hypersensitivity

This survey is funded by the Food Standards Agency. The survey is voluntary, and you are free to exit at any point - you don’t need to answer all the questions.

What will the survey involve?

This survey asks about your experiences and how you think the FSA can help people affected by food hypersensitivity to make safe food choices. Note that the FSA is not responsible for the diagnosis or management of food hypersensitivity.

We will use your responses to help the FSA define and prioritise its research activities in the area of food hypersensitivity. It will take approximately 10 minutes to complete. If you have any problems completing this survey, please email fsadigital@food.gov.uk. We will not ask you for any personal data;

The only personal details that we will be collecting are: your age range; whether you live in the UK; and your general demographic i.e. consumer, business, charity etc. This is so we can ensure we hear from a broad range of people. You will not be identifiable from this information. Please do not include any other personal details in your answers.

For further information on how FSA handles the information you have shared with us, please see our privacy policy on our website https://www.food.gov.uk/about-us/privacy-policy

Questions

Theme: Eating Out

Eating out describes the consumption of food away from home, especially at a restaurant, café or take away establishment.

Thinking about the experience of eating out, what unanswered questions and/or issues should the FSA try to answer in order to help people with food hypersensitivity?

Theme: Buying Prepacked Food

Prepacked food describes food that has been prepared in advance of sale e.g. ready meals, packaged sandwiches etc.

Thinking about the experience of buying prepacked food from shops, what unanswered questions and/or issues should the FSA try to answer in order to help people with food hypersensitivity?

Theme: Handling and Understanding Food

Handling and understanding food means being able to make informed choices about buying safe food, which involves: food preparation, labelling, food/ingredients supply, preventing cross-contamination, effective cleaning, testing and monitoring to ensure food safety.

Thinking about the experience of handling and understanding food, what unanswered questions and/or issues should the FSA try to answer in order to help people with food hypersensitivity?

Theme: Changes in how we interact with food

This relates to changes in how and where we obtain food today e.g. new foods and novel allergens, food banks, food business practices, new and reusable packaging, online purchasing through the internet etc.

Thinking about changes in the food we eat and where we get it from, what unanswered questions and/or issues should the FSA try to answer in order to help people with food hypersensitivity?

Theme: Improving what we know about food allergy and food hypersensitivity
What unanswered questions and/or issues about food hypersensitivity should the FSA try to answer, in order to help ensure that food is safe for people with food hypersensitivity?

For example, your questions could be about the numbers of people in the UK affected by food hypersensitivity; or why some people develop food hypersensitivity but then outgrow their allergy or sensitivity.

Demographic Questions

Do any of the following apply to you?

- I am a member of the general public with an interest in food hypersensitivity
- I have a food hypersensitivity myself
- I care for someone with a food hypersensitivity and /or I am completing this survey on behalf of someone else affected by food hypersensitivity e.g. my child
- I am a food business operator, representative or work for a food business
- I work or volunteer for a charity who helps provide for people with food hypersensitivities
- I work for a local authority or professional body with an interest in food hypersensitivity
- I am a healthcare professional (e.g. doctor, nurse, dietitian etc)
- I am a researcher with an interest in food hypersensitivity
- Other (free text)
- Prefer not to say

Do you currently live in the UK?

- Yes
- No

What age are you?

- 18-24
- 25-34
- 35-44
- 45-54
- 55-64
- 65-74
- 75+

Table S1: Indicative themes and sub-questions

**1. What is the type and prevalence of food hypersensitivity?**
How many people are affected by FHS?
How many hospital and/or doctors visits are caused by FHS incidents?
Is FHS increasing?
What are the most common allergies/intolerances?
What is the difference between an allergy and intolerance? (risk communication to public)
Are people being treated for symptoms of an allergy and not the cause?
**2. How and why do people develop food hypersensitivity?**
Why do people develop FHS?
Microbiome/microbiota and allergy development
Why do you get more allergies as you get older?
What factors make it more likely that you will get FHS?
Can you grow out of FHS? / does sensitivity/severity change with age?
Are allergies changing as our foods change?
Impact of behaviours on reaction severity
Impact of co-factors on reaction severity (in scope)
What is known about the link between development of FHS and their environment? (a lot of research has been carried out on this topic)
Is there a link between eczema in childhood and developing an allergy? (research has been conducted/is this fully addressed?)
Is there a link people who have food poisoning and then getting allergies/FHS? (interesting links to intolerance)
**3. Is it necessary to review the regulatory allergens list?**
What other allergenic ingredients exist?
Should the allergen list go beyond the EU 14?
What foods are cross reactive with other allergens/intolerance?
What is the risk of ingredients derived from allergens?
Is it possible to legislate/regulate for unexpected ingredients e.g. pea protein?
Do novel food processing techniques make foods more allergenic?
Can products used during growing of foods cause FHS? (only in scope if related to pesticides/residues left on food)
**4. What is the role of FODMAPS in trigging IBS?**
What is known about the role of FODMAPS in triggering IBS?
**5. What do the general public understand about FHS**
What do the general public know about FHS?
Should educate children so they understand FHS
How can we increase understanding of FHS amongst the general public?
**6. What information do consumers with FHS need to make choices about food?**
Do consumers feel safe to eat out?
How confident are consumers about traceability of food and integrity of supply chain?
Would more regulation satisfy consumers' concerns about eating out choices?

How do people with FHS know if staff are properly trained in managing/responding to FHS?
**7. What is the most effective way of communicating information?**
What would be the most effective way to communicate with consumers with FHS?
How can people report a food allergen incident?
How can consumers report breaches?
**8. What do businesses understand about FHS?**
Do food businesses understand FHS?
How do businesses reduce cross contamination?
How do businesses manage traceability, how confident are they in the accuracy of that information?
**9. How effective is monitoring and enforcement?**
Is it possible to combine hygiene ratings with a standard rating for allergies?
Can competence be measured via food safety/HACCP?
What testing is done on establishments to monitor processes to avoid cross contamination?
What testing is done to measure levels of knowledge of FHS amongst business operations?
Can there be a certification for businesses trained in FHS?
How many novel/online takeaways/street vendors have allergy information, can there be better regulation of businesses?
How to improve staff training?
What testing is done to ensure compliance with food regulations?
**10. How effective are existing measures/guidance on reducing cross contamination?**
What controls are needed to cook gluten free and gluten-containing foods in the same oven without cross contamination?
What food preparation needs to be in place to reduce cross contamination and guarantee something is free from a FHS ingredient?
Risk of cross contamination for coeliac disease e.g. fried food and how to mitigate
How can traceability be improved to monitor cross contamination? / How do businesses monitor traceability?
Could assessments be introduced so cross contamination is accurately assessed?
Risk posed by latex gloves
**11. How effective is guidance on producing gluten free food?**
Is there centralised guidance for manufacturers to ensure they are producing gluten free food? (work is being done on this)
**12. What improvements are required to current labelling of prepacked food?**
Is labelling clear enough? Use of symbols/icons rather than (or in addition to) words? How to display ingredients clearly on mixed/multipack foods
Is listing of allergen traces in prepacked food helpful?
Could similar labelling be in place for FHS as there are for allergens?
Can E-numbers be translated to allergens e.g.E322 soya? (possibly relates to lack of consumer understanding)
Can labels highlight when ingredients change?
How many people rely on information other than the food packaging label?
Labelling to highlight actual risk of cross contamination (ingredients/packaging)

What is the risk from trace levels?
**13. What improvements are required to current labelling of non-prepacked food?**
Full allergens should be listed on the menu
Menus include advice about whether food can be adapted to exclude allergens
Regulation forcing FBOs (inc. street vendors/takeaways) to label foods correctly
Labelling to highlight actual risk of cross contamination (ingredients/packaging)
**14. What role can digital technology play in providing information to consumers/businesses in the future?**
Can digital technology be used as a better means of displaying allergen info?
How reliable are food apps for providing information?
What level of detail is needed to communicate allergen risk?
**15. What regulations/guidance is needed for novel packaging?**
What testing is being done on novel packaging?
**16. What regulations/guidance is needed for package free shopping and reusable packaging?**
What impact will package free shopping have on those with FH? (relates to labelling and cross contamination)
**17. Is there an advantageous commercial value to providing safe food to consumers with FHS?**
Is there any evidence that businesses benefit economically when they cater for consumers with FHS?
Could food producers be incentivised to exclude FHS ingredients to increase consumer choice?
What impact does socio-economic factors have on consumers with FHS? (e.g. availability of gluten-free food/allergen free food in food banks)

Table S2: Indicative uncertainties identified by IPSOS MORI analysis

Initial indicative question	Questions for prioritisation	Rationale
**What is the type and prevalence of food hypersensitivity?**	none	Existing FSA-commissioned research is examining this:<br>• PAFA study (adults)<br>• Anaphylaxis (all ages)
**How and why do people develop food hypersensitivity?**	What are the environmental exposures which increase the risk of developing food hypersensitivity?<br><br>How do socio-economic factors impact on individuals with food hypersensitivity?<br><br>What co-factors (other than exercise and sleep deprivation) increase the risk of a severe allergic reaction?	Responses highlighted whether individuals from some SE backgrounds are at greater risk of severe FHS reactions
**Is it necessary to review the regulatory allergen list?**	none	Existing FSA-commissioned research is examining this (anaphylaxis register)
**What is the role of FODMAPS in triggering IBS?**	none	Not in FSA remit
**What do the general public understand about food hypersensitivity?**	What is the current level of existing knowledge of food hypersensitivity in the general public (including consumers and food business operators), and how can this be improved?	
**What information do consumers with food hypersensitivity need, to make choices about food?**	What is the most effective way to alert consumers as to changes in ingredients?<br><br>What are the best ways to communicate risk and appropriate mitigating actions to consumers and food business operators (including, but not limited to, online food	

**What is the most effective way of communicating information to consumers with food hypersensitivity?**	business operators/street vendors / food banks)?<br><br>What are the risks posed due to shared production (e.g. cooking) of foods to individuals with food hypersensitivity, and how can these be mitigated?<br><br>What are the best ways for consumers to be confident that the food they are provided with is safe with regard to food hypersensitivity?<br><br>How can food business owners improve traceability of allergens in the food supply chain	
**What do businesses understand about food hypersensitivity?**	What is the current level of existing knowledge of food hypersensitivity in the general public (including consumers and food business operators), and how can this be improved?<br><br>What are the risks posed due to shared production (e.g. cooking) of foods to individuals with food hypersensitivity, and how can these be mitigated?<br><br>What risk is posed to health by "derived" ingredients such as plant oils /fats, starches (other than those foods for which legal exemptions currently exist)?<br><br>How can food business owners improve traceability of allergens in the food supply chain	The sensitivity and reliability of analytical tests was also discussed, but research/development of these and the responsibility to ensure such tools are used appropriately was felt to be outside the FSA's research remit.
**How effective is monitoring and enforcement?**	What is the best way to alert the FSA as to incidents involving food hypersensitivity (and increase awareness of how to do this amongst the general public)?	This is captured through existing incidents data

**How effective are existing measures/guidelines on reducing cross contamination?**	What are the risks posed due to shared production (e.g. cooking) of foods to individuals with food hypersensitivity, and how can these be mitigated?<br><br>What is the risk to latex-sensitive consumers from latex cross-contact (both via packaging and during food handling)?	
**How effective is guidance on producing gluten free food?**	What are the risks posed due to shared production (e.g. cooking) of foods to individuals with food hypersensitivity, and how can these be mitigated?	Food and Drink Federation and Coeliac UK produced evidence-informed guidance to the food industry in 2019. Coeliac UK has undertaken research on risks of gluten cross-contamination in the kitchen environment.
**What improvements are required to current labelling on prepacked food?**	What are the best ways to communicate risk and appropriate mitigating actions to consumers and food business operators (including, but not limited to, online food business operators/street vendors / food banks)?<br><br>What do consumers want from allergen labelling, including Precautionary Allergen (e.g. "may contain") Labelling)?	
**What improvements are required to current labelling of non-prepacked foods sold via food business operators?**	What are the best ways to communicate risk and appropriate mitigating actions to consumers and food business operators (including, but not limited to, online food business operators/street vendors / food banks)?<br><br>What do consumers want from allergen labelling, including Precautionary Allergen (e.g. "may contain") Labelling)?	

**What role can digital technology play in providing information to consumers and FBOs?**	What is the best way to alert the FSA as to incidents involving FHS (and increase awareness of how to do this amongst the general public)?<br><br>How can food business owners improve traceability of allergens in the food supply chain?<br><br>What are the best ways to communicate risk and appropriate mitigating actions to consumers and food business operators (including, but not limited to, online food business operators/street vendors / food banks)?	
**What regulation/ guidance is needed for novel packaging?**	What risk is posed to people with food hypersensitivity by new/novel foods and/or processes (including packaging and other food contact materials)?	
**What regulation/ guidance is needed for package free and reusable packaging?**		
**Is there commercial advantage to providing for consumers with food hypersensitivity?**	Are there economic benefits to providing for individuals with food hypersensitivity, which can be used to encourage food business operators to go beyond the letter-of-the-law?	
